# Predicting the genomic resolution of bulk segregant analysis

**DOI:** 10.1093/g3journal/jkac012

**Published:** 2022-02-07

**Authors:** Runxi Shen, Philipp W Messer

**Affiliations:** Department of Computational Biology, Cornell University, Ithaca, NY 14853, USA

**Keywords:** QTL mapping, coalescence theory, experimental design

## Abstract

Bulk segregant analysis is a technique for identifying the genetic loci that underlie phenotypic trait differences. The basic approach is to compare two pools of individuals from the opposing tails of the phenotypic distribution, sampled from an interbred population. Each pool is sequenced and scanned for alleles that show divergent frequencies between the pools, indicating potential association with the observed trait differences. Bulk segregant analysis has already been successfully applied to the mapping of various quantitative trait loci in organisms ranging from yeast to maize. However, these studies have typically suffered from rather low mapping resolution, and we still lack a detailed understanding of how this resolution is affected by experimental parameters. Here, we use coalescence theory to calculate the expected genomic resolution of bulk segregant analysis for a simple monogenic trait. We first show that in an idealized interbreeding population of infinite size, the expected length of the mapped region is inversely proportional to the recombination rate, the number of generations of interbreeding, and the number of genomes sampled, as intuitively expected. In a finite population, coalescence events in the genealogy of the sample reduce the number of potentially informative recombination events during interbreeding, thereby increasing the length of the mapped region. This is incorporated into our model by an effective population size parameter that specifies the pairwise coalescence rate of the interbreeding population. The mapping resolution predicted by our calculations closely matches numerical simulations and is surprisingly robust to moderate levels of contamination of the segregant pools with alternative alleles. Furthermore, we show that the approach can easily be extended to modifications of the crossing scheme. Our framework will allow researchers to predict the expected power of their mapping experiments, and to evaluate how their experimental design could be tuned to optimize mapping resolution.

## Introduction

The advent of easy and affordable genome sequencing has enabled powerful genetic mapping approaches. In addition to advancing our understanding of the molecular basis of phenotypic traits, such approaches can have important practical applications. For example, genetic mapping can help us identify variants that underlie human diseases (Altshuler et al. 2008), localize genes associated with favorable traits in plant or animal breeding (Womack 1997; Goddard and Hayes 2009), and detect the loci responsible for drug or pesticide resistance (Ranson et al. 2000, 2004; Anderson et al. 2011).

Various techniques have been developed for this purpose, ranging from classical linkage mapping to genome-wide association studies (GWAS), with numerous extensions or combinations of these approaches that are often tailored toward specific applications. Which particular technique is best suited for a given problem can depend on a variety of factors, such as the genetic architecture of the trait, the specific biology of the study system, the resources available for experiments and sequencing, and the mapping resolution desired.

In species that can be experimentally crossed, classical linkage mapping has proven a powerful technique for detecting quantitative trait loci (QTL) (Paterson et al. 1988; Lynch and Walsh 1998; March 1999; Zeng 2001). One example of classical linkage mapping is backcross mapping, which involves the generation of an F_1_ cross from two parental strains of contrasting phenotypes. The F_1_ offspring are then backcrossed to the parental strains, and the resulting progeny are phenotyped for the trait of interest and genotyped at a set of marker loci distributed across the genome. By scanning for markers with an inheritance pattern that correlates with the trait, one can localize the segments of the genome on which causal variants could reside. This method has long been the primary genetic mapping technique, yet it tends to attain rather low genomic resolution (i.e. the length of the identified genomic region in which the causal locus must be contained but cannot be more precisely pinpointed). This is because the segments linked to the parental strains are typically quite long due to the limited number of recombination events in a single cross.

GWAS is an alternative approach for QTL mapping in which a large number of individuals from a genetically diverse population are genotyped at a dense set of SNP markers, or by whole genome sequencing, and phenotyped for the trait of interest (Visscher et al. 2017). The QTL responsible for trait variation can then be identified by regressing SNP genotypes against the phenotype. The genomic resolution of this approach is limited in principle only by the density of SNP markers and the genomic distance over which linkage disequilibrium decays in the mapped population. As a result, GWAS can sometimes detect even individual causal SNPs. However, the trait of interest needs to exhibit sufficiently high levels of additive genetic variation for GWAS to work, and detection power tends to be limited for causal variants that segregate at low population frequency. In addition, due to the large number of SNPs tested, the thresholds for calling statistical significance can be quite high.

Bulk segregant analysis (BSA) is a mapping approach that combines ideas from linkage mapping and GWAS (Michelmore et al. 1991). Like classical linkage mapping, a typical BSA design starts from two parental strains of contrasting phenotypes. These strains are then crossed to generate an F_1_ population that is further interbred for several generations while maintaining a sufficiently large population size to allow recombination to break up linkage from the two parental strains. In the final generation, two pools of individuals are selected from the tails of the phenotypic distribution, and each of these pools is sequenced. The alleles responsible for trait differences (as well as any alleles linked to them) should then exhibit significant frequency differences between the two pools, while alleles at other loci should be present in both pools at similar frequencies.

In contrast to both GWAS and classical linkage mapping, BSA does not require the sequencing of individual genomes, since only the overall allele frequencies in the two pools are relevant. This allows the use of more economic sequencing approaches such as Pool-seq (Schlötterer et al. 2014). The resolution of BSA is still expected to be considerably lower than GWAS because the number of generations over which the population is interbred will be limited. For longer experiments, the effects of drift could also become problematic (Pool 2016). However, BSA can still be used for detecting QTL where causal alleles are segregating at low frequency in the population, as long as they are present in one of the parental strains. This could be an important factor for applications such as the mapping of drug or pesticide resistance mutations.

Conceptually, BSA is similar to “introgression mapping” (Severin et al. 2010; Earley and Jones 2011), where the population is repeatedly selected for the phenotype of the first parental strain in every even generation of the experiment. The surviving individuals are then back-crossed to the second parental strain and the resulting offspring are interbred without selection in every odd generation. Under this approach, the population at the end of the experiment should be genetically similar to the first parental strain in the genomic regions that surround causal QTL, while it should be similar to the second parental strain for the rest of the genome. Note, however, that this approach can require a considerably higher experimental effort than BSA.

BSA has already been successfully applied in various contexts. For example, implementations of this approach have been used to identify DNA markers linked to disease-resistant genes in lettuce (Michelmore et al. 1991) and pest-resistant genes in crops (Snoeck et al. 2019), to study horizontal gene transfer in *Tetraychus urticea* (Bryon et al. 2017), to locate QTLs associated with drought resistance in maize (Quarrie et al. 1999), and to map the genetic basis of various complex traits in yeast and *Drosophila* (Lai et al. 2007; Ehrenreich et al. 2010; Magwene et al. 2011).

Despite these successful applications, one practical shortcoming of BSA is that, depending on the experimental design, it tends to produce very wide peaks of significance, which in previous studies have sometimes extended over hundreds of kilobases (Wybouw et al. 2019) or even several megabases (Song et al. 2017). This is particularly problematic because we do not currently have a good understanding of how the expected mapping resolution is determined by biological and experimental parameters. Simulation studies have shed some light on this issue and demonstrated that more generations of interbreeding, a larger population size during interbreeding, and deeper sequencing can all improve mapping resolution, while the size of the selected pools apparently has less of an impact (Pool 2016). Nevertheless, it would still be desirable to have an analytical understanding of exactly how all of these factors influence mapping resolution; this would allow us to predict the expected resolution for a given experiment, and to assess which factors one should tune to optimize the mapping resolution most economically.

In this study, we employ coalescence theory to develop an analytical framework for calculating the expected mapping resolution of a BSA experiment. Our results reveal how the recombination rate of the study organism, the effective population size during interbreeding, the overall length of the experiment, and the number of genotyped individuals combine to determine the maximally achievable mapping resolution for a trait with a simple genetic architecture.

## Materials and methods

### Simulations of BSA experiments

Simulations of BSA experiments were implemented in the SLiM evolutionary simulation framework (version 3.5) (Haller and Messer 2019). We modeled a single QTL located on a 100-Mb-long chromosome. Each experiment was initialized with two homozygous parental strains (denoted as *AA* and *aa* strains). The F_1_ was always seeded with 1,000 males from the *AA* strain and 1,000 females from the *aa* strain. The population was then interbred over *t* discrete, nonoverlapping generations, using SLiM’s default Wright–Fisher model without selection. While the total number of individuals was kept constant at 2,000 in each generation, only *N_e_* randomly chosen individuals were actually allowed to mate and reproduce in each generation. Recombination occurred at a uniform rate of r=1e−8 per bp along the chromosome (corresponding to 1 cM/Mb) in all simulations.

For the comparisons of analytical vs simulation results in the standard BSA design (Fig. 5), as well as the heterozygote selection (HS) scheme (Fig. 6), we used SLiM’s tree-sequence recording feature (Haller et al. 2019) to track the ancestry at each position in each genome. This allowed us to directly identify ancestry breakpoints (defined in *Results*) in the sampled chromosomes without having to model any marker SNPs for such inference.

For the simulations of the introgression mapping (IM) scheme (Fig. 6), we modeled SNPs placed at equidistant intervals of 10 kb along the chromosome to differentiate ancestry from the two parental strains. While this approach only allows for indirect and approximate inference of ancestral breakpoint locations, it should not pose a limiting factor given that the mapping resolution was typically several orders of magnitude larger than the distance between marker SNPs.

For the simulation of a short-read Pool-seq experiment (Fig. 7), we used marker SNPs placed at equidistant intervals of 1 kb along the chromosome. Here, we assumed that every SNP provided an independent locus where a new set of *C* chromosomes was genotyped from the 2*s* chromosomes present in each sample. These chromosomes were chosen randomly with replacement.

The SLiM models for simulations, Python scripts for data analysis, and all other relevant files are available at: https://github.com/runxi-shen/Predict-Genomic-Resolution-of-BSA (last accessed January 23, 2022). We also provide a simple python script *predict_bsa_resolution.py* for calculating the expected genomic resolution of a BSA experiment according to our equations, given the experimental design and parameters. A detailed documentation of these scripts and instructions for how to use them for different experimental designs are explained in the file *REAME.md*, which is also provided on the Github repository.

### Calculation of G′

We calculated G′ from SNPs placed at equidistant intervals of 10 kb (or 1 kb for the Pool-seq experiments) along the chromosome to differentiate ancestry from the two parental strains. These calculations followed the procedure described in (Magwene et al. 2011). Smoothed curves were obtained by a weighted sum of all SNPs within the window bracketing the focal SNP, where the weight of each SNP was obtained by a Nadaraya–Watson kernel regression (Watson 1964). For peak calling in Figs. 7 and 8, we used the 99.9th percentile of all G′ values along the chromosome as the significance threshold (Zhang and Panthee 2020). All SNPs with G′ values above this threshold were considered part of the peak, with the size of the peak determined by the distance between its leftmost and rightmost SNPs.

## Results

**Fig. 1.**
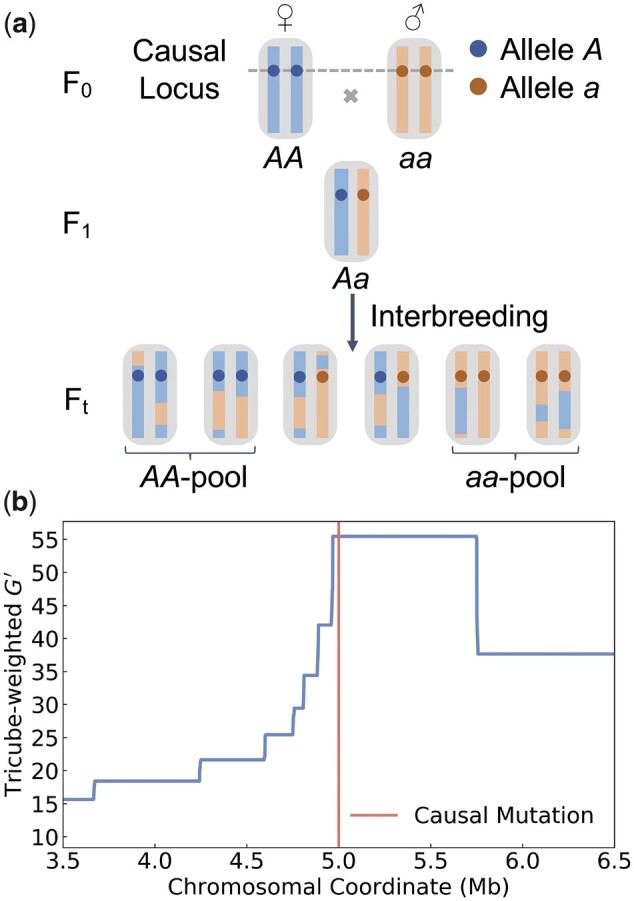
Illustration of a BSA experiment. a) Our model assumes a trait determined by a single QTL with two different alleles (*A*, *a*). The starting point of the experiment are two inbred parental strains, represented by red and blue chromosomes. The blue strain carries the *A* allele and the red carries the *a* allele. An F_1_ population is created and interbred for *t* generations. At the end of the experiment, two pools of individuals are selected such that the first comprises only *AA* individuals and the second only *aa* individuals. The mapping resolution is determined by the length of the region surrounding the QTL for which all chromosomes in the *AA*-pool still have blue ancestry, while all in the *aa*-pool still have red ancestry. b) Mapping resolution in a simulated BSA experiment for a QTL located at the center (red line) of a 10-Mb-long chromosome (only showing the genomic segment between 3.5 and 6.5 Mb in figure). Interbreeding was modeled for 10 generations in a population of 100 individuals with a uniform recombination rate of 1.0 cM/Mb. Two pools of 10 *AA* and 10 *aa* individuals were selected at the end of the experiment. The blue curve shows the G′ statistic estimated from marker SNPs. The peak in G′ around the QTL indicates the region where all chromosomes in the *AA*/*aa* pools still have blue/red ancestry, which extends for ∼0.5 Mb.

To develop a theoretical understanding of the expected mapping resolution of a BSA experiment, it will be instructive to first consider a highly idealized model of a phenotypic trait determined by a single QTL which we assume has two segregating alleles: *A* and *a*. We further assume that recombination occurs at a uniform rate *r* per bp along the chromosome, which we model by a Poisson process. We neglect gene conversion and assume that recombination events always result in crossover. Starting from the two parental inbred strains (“blue” and “red”) which we assume have genotypes *AA* and *aa* at the QTL, a BSA experiment is performed for *t* generations of interbreeding, as outlined in Fig. 1a.

At the end of the experiment, we select two samples from the interbred population, such that the first sample contains *s* chromosomes of genotype *A*, while the second contains *s* chromosomes of genotype *a*. This could be achieved, for example, by selecting s/2 individuals that are homozygous for *A* as the first sample, and another s/2 individuals that are homozygous for *a* as the second sample if we can accurately identify such homozygotes based on their phenotype alone. Each of the two samples is then sequenced individually. Note that this may require the phenotyping of many more than *s* individuals in an actual experiment (thus, the size of the phenotyped population could be substantially larger than the bulk size). Furthermore, there will typically be some level of contamination of the segregant pools with alternative alleles (e.g. due to phenotyping errors, incomplete heritability, or when heterozygotes cannot be reliably distinguished from the homozygotes by phenotype alone). However, it will nonetheless be instructive to study an idealized model with uncontaminated pools first. We will then investigate below how results are affected by different levels of contamination.

As a consequence of recombination during interbreeding, each of the chromosomes sampled at the end of the experiment should be a mosaic of red and blue ancestry segments. However, there should be a region surrounding the QTL where all chromosomes in the *AA*-pool still have blue ancestry, while all in the *aa*-pool still have red ancestry. The maximally achievable mapping resolution is determined by the size of this region (assuming that there is only one such region in the sample). Note that in real-world experiments the ancestry breakpoints will not be directly observable. Instead, their location can only be inferred approximately through marker SNPs that allow one to distinguish ancestry from the two founding strains. The genomic density of such marker SNPs thus places a practical limit on the achievable mapping resolution; however, this should not be problematic as long as the average distance between differentiating sites remains short compared to the mapping resolution predicted by our calculations.

Several summary statistics have been developed to identify these regions of contrasting ancestry between the segregant pools, typically based on the detection of allele frequency differences at marker SNPs. Examples for such statistics include ancestry difference (*A_d_*) (Pool 2016), Δ(SNP-index) (Fekih et al. 2013), and a modified *G*-statistic (G′) (Magwene et al. 2011). An illustration of this mapping problem is provided in Fig. 1b, where we show G′ estimated along a chromosome in a simulated BSA experiment.

**Fig. 2.**
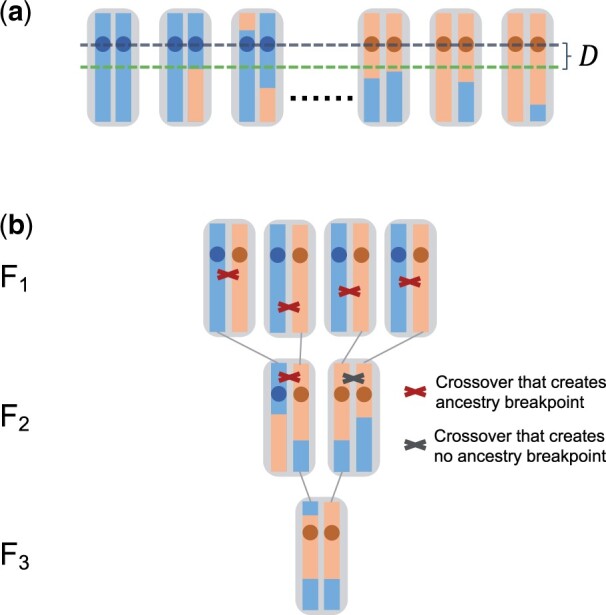
Resolution of a BSA experiment. a) We define *D* as the distance between the QTL and the first ancestry breakpoint downstream of the QTL in all samples. b) Example of a full pedigree of an individual from the F_3_. All crossover events that have occurred along its pedigree are also shown. Only those crossover events occurring in individuals that carried red and blue ancestry at the location of the crossover actually generated new ancestry breakpoints, and every breakpoint observed in the sampled chromosome can be traced back to such a specific crossover event in the pedigree.

The goal of our theoretical analysis will be to calculate the expected length of the region where all chromosomes in the *AA*-pool still have blue ancestry, while all in the *aa*-pool still have red ancestry. For this purpose, let us define *D* as the distance to the closest “ancestry breakpoint” (defining a point where ancestry changes between blue and red in a chromosome) located downstream of the QTL among all chromosomes in the samples (Fig. 2a). Due to symmetry, the expected length of the mapped region will then be simply 2E[D], where E[D] denotes the expectation value of *D* (we will neglect edge effects when a QTL is located close to the start/end of the chromosome). This length determines the expected mapping resolution of the BSA experiment (with “shorter” expected mapping tract lengths corresponding to “higher” resolution). Note that the actually achievable resolution will likely be lower in practice than predicted by our theory due to the need to rely on marker SNPs as proxies for ancestry, as well as other experimental factors such as sequencing errors.

Our general approach for the calculation of E[D] is to trace the lineages of all sampled chromosomes back to the two parental strains, and then study how ancestry breakpoints have been generated along this genealogy (Fig. 2b). Note that due to recombination events, local genealogies will vary as one moves along the chromosomes of the samples, constituting the so-called “tree sequence” (Kelleher et al. 2016). However, at any given position, there will be exactly one genealogy. Thus, the lineage of any given sampled chromosome at that position can be traced back all the way to a single chromosome in the F_0_. If this happens to be a red chromosome, the sampled chromosome will be assigned red ancestry at this position, otherwise it will be assigned blue ancestry.

Each ancestry breakpoint in a sampled chromosome stems from a crossover event in one of its ancestors. Importantly, this must have been an ancestor that carried a blue ancestry segment around the crossover location in one of its chromosomes, and a red one in the other (Fig. 2b). By contrast, crossover events at positions where an individual carries either two blue or two red ancestry segments around the crossover location will never create new ancestry breakpoints.

### Infinite population model

We initially want to assume an idealized model of an interbreeding population of infinite size. This is for two reasons: first, we want to be able to neglect coalescence events when tracing back the lineages from the chromosomes in our sample to the chromosomes in parental strains. Second, we want to be able to neglect any changes in allele frequencies over the course of the experiment due to random genetic drift.

Let us first consider a short BSA experiment where the population is already sampled in the F_2_. Since all individuals in the F_1_ carry one red and one blue chromosome, all recombination events in this generation should create new ancestry breakpoints. We model a uniform recombination rate (*r*) per base pair. In 2*s* sampled chromosomes from the F_2_ (representing the combined two pools), the overall rate (*R*) at which new ancestry breakpoints have been created per bp in this generation is therefore simply R=r×2s.

Assuming R≪1, we can model these events by a Poisson process along the chromosome. The distance *D* to the closest ancestry breakpoint downstream of the QTL in all sampled chromosomes should then be an exponential random variable with cumulative density function $P(D\leq d)=1-e^{-Rd}$ and expectation value E[D]=1/R=1/(2rs).

We can directly extend this process to chromosomes sampled from the F_3_, but here things become a bit more complicated. This is because the parents of the sampled individuals are no longer guaranteed to carry one red and one blue chromosome. Instead, according to Hardy–Weinberg equilibrium, the probability that a randomly picked individual from the F_2_ at any given genomic position will carry chromosomes with different ancestry is only 1/2. Thus, only half of the crossover events during meiosis are actually expected to create new ancestry breakpoints in this generation, and the overall rate at which new ancestry breakpoints are created per bp is therefore *R* = *rs*. Since we neglect drift in the infinite population model, this should be the same fraction for all future generations.

The infinite population model also ensures that no two sampled chromosomes will ever share a parent or grandparent with each other. Consequently, we can model individual ancestral lineages completely independently of each other. In 2*s* chromosomes sampled from the F_3_, the overall rate (*R*) at which new ancestry breakpoints have been generated per bp is therefore simply the sum of the individual rates over the 2*s* lineages and the two parental generations: R=2rs+rs=3rs. Every additional generation of crossing will further increment this rate by *rs*. Thus, after *t* generations of interbreeding, the overall rate will be *R* = *rst*. Assuming R≪1, we can again model these events by a Poisson process along the chromosome, yielding:

(1) $$E[D]=\frac{1}{R}=\frac{1}{\mathrm{rst}}.$$

Because, the situation upstream and downstream of the QTL is symmetric, the expected resolution of the BSA experiment in this infinite population mode is then simply 2E[D]=2/(rst) bp. Thus, it is inversely proportional to the product of the recombination rate, sample size, and length of the experiment. This result is very intuitive; all that matters is the overall rate at which new ancestry breakpoints are generated along the lineages of the sample.

### Finite population model

**Fig. 3.**
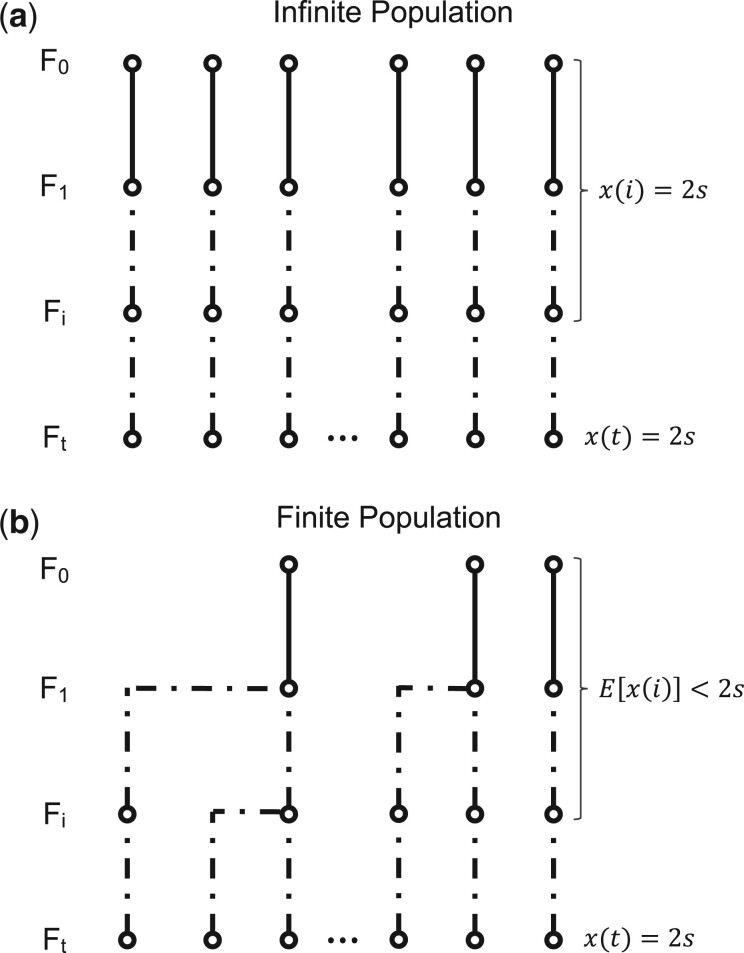
Infinite and finite population models. a) In the infinite population model, all lineages descend independently and the overall length of the genealogy of 2*s* sampled genomes is simply 2*st*. b) In the finite population model, by contrast, lineages can coalesce in ancestors of the sample, reducing the expected overall number of ancestors in previous generations and thereby the expected length of the genealogy.

In the infinite population model, every ancestry breakpoint present in the sampled chromosomes traces back to a unique crossover event along the genealogy of the sample. In a finite population, different chromosomes can share a breakpoint that traces back to the same crossover event in a common ancestor. In that case, we can no longer describe the genealogy of the samples by 2*s* distinct lineages through the interbreeding phase, since individual lineages could have merged (Fig. 3).

An important consequence of this is that the average “length” of the sample’s genealogy will be shorter in a finite population compared to our infinite population model, where it was simply 2*st*. In general, this should reduce the number of ancestry breakpoints captured in the sample, thereby increasing the length of the mapped region.

To derive an analytic expression for the mapping resolution in a finite population, let us assume that we can model it as a diploid Wright–Fisher population with coalescence effective population size *N_e_*. Let *x*(*i*) denote the number of ancestral lineages at a given genomic position in the genealogy of the sampled chromosomes in generation *i* (Fig. 3). We can calculate how *x*(*i*) is expected to change between consecutive generations, applying a result from the theory of occupancy distributions (Johnson and Kotz 1977):

(2) $$E[x(i-1)]=2N_{e}\left[1-{\left(1-\frac{1}{2N_{e}}\right)}^{x(i)}\right].$$

Evaluating this equation recursively, starting from x(t)=2s, then allows us to calculate the expectation values of *x*(*i*) all the way back to *i *=* *2.

As in the infinite population model, every crossover event in the F_1_ will create a new ancestry breakpoint, while this should be true for only half of such events in subsequent generations. Together with the above result for *x*(*i*), this allows us to calculate the overall rate (*R*) at which new ancestry breakpoints are generated per bp along the genealogy of all sampled chromosomes:

(3) $$R=rx(2)+\frac{r}{2}\sum_{i=3}^{t}x(i).$$

Since E[x(i)]<2s for all *i *<* t*, this rate will be smaller than the corresponding rate *R* = *rst* of the infinite population model.

One important assumption underlying Eq. (3) is that the population frequencies of red and blue alleles still remain constant at 50% over the course of the experiment, so that from the F_2_ onward, the probability that a randomly chosen individual carries both a red and a blue ancestry segment at any given genomic position remains at 0.5. However, random genetic drift should lead to a decay of heterozygosity (*H*) over time according to $H\propto \;\text{exp}(-t/(4N_{e})]$, and the probability that an individual carries ancestry segments from both parental strains at a given genomic position is expected to decrease at a similar rate. Since we neglect this effect, Eq. (3) should still overestimate *R*, although much less so than in the infinite population model. This should primarily be a problem for very long BSA experiments with small *N_e_* where $t\ll 4N_{e}$ does not hold.

As long as R≪1, we can again model the creation of new ancestry breakpoints by a Poisson process along the chromosome. The distance *D* to the closest ancestry breakpoint downstream of the QTL captured in the sample will then be an exponential random variable with expectation value:

(4) $$E[D]=\frac{1}{R}=\frac{1}{rx(2)+\frac{r}{2}\sum_{i=3}^{t}x(i)}.$$

This result provides an analytic solution for the expected mapping resolution of a BSA experiment with an interbreeding population of effective size *N_e_*. However, its calculation requires iterative evaluation of Equation (2), and we are not aware of any closed-form solution for this recursion. Even though all elements of *x*(*i*) can be easily calculated with the help of a computer, this may not be particularly helpful in allowing us to understand how individual parameters are expected to affect the mapping resolution. To address this issue, we will make use of a previously suggested deterministic approximation for *x*(*i*), which can be obtained by mapping the recursion to a differential equation (Griffiths 1984; Maruvka et al. 2011; Chen and Chen 2013; Jewett and Rosenberg 2014):

(5) $$x(i)\approx \frac{2s}{2s-(2s-1)e^{-\frac{t-i}{4N_{e}}}}.$$

We will further replace the summation in Equation (3) by an integral over the *t* generations of the experiment, yielding:

(6) $$R\approx \frac{r}{2}\int_{0}^{t}x(y)dy=2N_{e}r\ln\left(2s\left[e^{\frac{t}{4N_{e}}}-1\right]+1\right).$$

Note that this integration assumes that recombination events along the genealogy create ancestry breakpoints with a uniform probability of 1/2 in every generation (not just from the F_2_ onward). This assumption is obviously incorrect for individuals in the F_1_, where every recombination event will generate a new ancestry breakpoint. However, by extending our integration back to the F_0_, where recombination events never generate new ancestry breakpoints, we effectively compensate for this effect, at least as long as E[x(0)]≈E[x(1)]. This yields an expected mapping resolution of:

(7) $$E[D]\approx \frac{1}{2N_{e}r\ln\left(2s\left[e^{\frac{t}{4N_{e}}}-1\right]+1\right)}.$$

In the following, we will refer to Equation (4) as the “recursion” solution, while the approximation presented in Equation (7) will be referred to as the “integration” solution.

### Limiting cases

We now want to take a closer look at the expected mapping resolution derived in Equation (7) and discuss how it relates to the result from the infinite population model. First, as we already mentioned above, our approach relies on the assumption that $t\ll 4N_{e}$, as drift would otherwise be strong and heterozygosity would be expected to decay noticeably over the course of the experiment. This assumption specifies a regime where the probability that a given pair of lineages coalesce over the course of the experiment is still small (since the expected time to pairwise coalescence should be $2N_{e}$ generations). Given $t\ll 4N_{e}$, we can perform a Taylor series approximation to the exponential in Equation (7):

(8) $$\begin{array}{c} \ln\left(2\left[e^{\frac{t}{4N_{e}}}-1\right]+1\right)\approx \ln\left(\frac{st}{2N_{e}}+1\right)\\ \Rightarrow E[D]\approx \frac{1}{2rN_{e}\ln\left(\frac{st}{2N_{e}}+1\right)}. \end{array}$$

This approximation allows us to better understand how the infinite and finite population models differ from each other. In the infinite population model, mapping resolution was simply inversely proportional to the product of recombination rate (*r*), sample size (*s*), and number of generations (*t*) of the experiment. In the finite population model, mapping resolution is still inversely proportional to the recombination rate, but the effects of sample size and experiment length are now attenuated by a logarithm. Consequently, increasing those parameters is no longer expected to improve mapping resolution as effectively as in the infinite population model. We further note that sample size and generations enter Equation (8) only in terms of the product *s *×* t*. Varying each of these two parameters by the same factor is therefore expected to produce a similar impact on the expected mapping resolution (as long as $t\ll 4N_{e}$ still holds). In practice, this means that running an experiment twice as long, for instance, should yield the same benefit as doubling the sample size.

Equation (8) also reveals where the effects of a finite population start to become substantial. When $st\ll 2N_{e}$, we can further approximate

(9) $$\ln\left(\frac{st}{2N_{e}}+1\right)\approx \frac{st}{2N_{e}}\Rightarrow E[D]\approx \frac{1}{\mathrm{rst}}.$$

Thus, the infinite and finite population models converge in this regime. The two models will increasingly diverge from each other as *st* becomes of the same magnitude as $2N_{e}$. The condition $st\ll 2N_{e}$ should typically be much stricter than $t\ll 4N_{e}$, our essential assumption for the finite population model, unless sample size is very small. The former effectively assumes that there are only very few coalescence events among the genealogy of all sampled chromosomes, whereas the latter only assumed that coalescence was unlikely between any two sampled chromosomes.

**Fig. 4.**
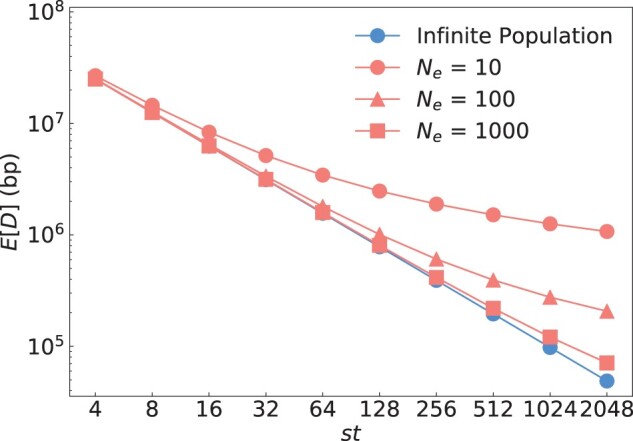
Mathematical predictions for the infinite and finite population models. Blue dots show the prediction by the infinite population model according to Equation (1); red dots show the prediction by the finite population model according to Equation (6) for three different values of *N_e_*. Recombination rate was set to r=1e−8 per bp. To vary *st* in these equations, we fixed *t *=* *2 and then varied *s* from 2 to 1,024. The infinite and finite population models converge as *st* becomes much larger than $2N_{e}$, as predicted by our theory.

Figure 4 illustrates the behavior of our analytical solutions for the finite and infinite population models as a function of the product *st*, and, in the finite population model, for different values of *N_e_*. As predicted, both models converge when $st\ll 4N_{e}$. Compared to the infinite population model, increasing *st* provides only diminishing returns for improving mapping resolution in the finite population model. Lower *N_e_* values generally decrease mapping resolution.

### Numerical validation

To evaluate the accuracy of our mathematical results, we conducted individual-based simulations of a BSA experiment (see *Materials and* *methods*). Specifically, we modeled an experimental setup as described in Fig. 1a, assuming a trait that is determined by a single QTL located on a 100-Mb-long chromosome. We assumed a uniform recombination rate of r=1e−8 per bp and generation (i.e. 1 cM/Mb), which we did not vary in our simulations because mapping resolution should always be inversely proportional to *r*. The parameters we did vary were the sample size (*s*), the effective population size (*N_e_*), and the number of generations of interbreeding (*t*).

**Fig. 5.**
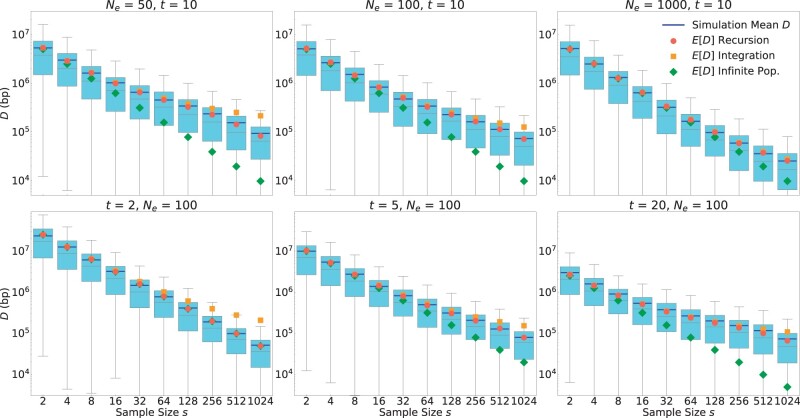
Comparison between mathematical results and simulations. A single QTL located on a 100-Mb-long chromosome with a uniform recombination rate of 1 cM/Mb was modeled. Box plots show the distribution (quartiles) of *D* estimated over 5,000 simulation runs for each given parameter setting. The top/bottom whiskers represent the highest/lowest datum within the 1.5 interquartile range of the upper/lower quartile. Blue lines show the means of the data, which tend to be much larger than the medians. Symbols show the expected resolutions for the infinite (green) and finite (red/orange) population models according to Equations (1), (4) and (7), respectively. Green dots are difficult to see in the lower left panel because they are almost completely overlaid by the red dots. Note that these mathematical predictions should be compared to the means of the simulations (blue lines), not the medians. In the top row, we varied *N_e_* while keeping *t *=* *10 constant. In the bottom row, we varied *t* while keeping *N_e_* = 100 constant.

Figure 5 shows the comparisons between these simulations and our analytical results given by Equations (1), (4), and (7) over a broad range of parameter values (*N_e_* varying between 10 and 1000, *s* varying between 2 and 1024, and *t* varying between 2 and 20). For each parameter setting, we estimated *D* over 5,000 simulations. The resulting distributions are shown by Box and Whisker plots. Note that these distributions tend to have rather pronounced positive skews, such that their means tend to be much larger than their medians. Our mathematical predictions are given in the form of expectation values for *D*, and thus need to be compared to the mean values of the simulation data, not the medians.

The simulation results show excellent agreement with our recursion solution for the finite population model provided in Equation (4). As already discussed above, the solution from the infinite population model provided in Equation (1) constitutes an upper bound for the maximally achievable mapping resolution. Consistent with analytical predictions, the finite and infinite models converge when $st\ll 4N_{e}$, and the infinite population model increasingly overestimates mapping resolution as the condition $st\ll 4N_{e}$ becomes violated.

The integration approximation of the finite population model we derived in Equation (7) generally works well for small and moderate sample sizes, but tends to underestimate mapping resolution when *s* approaches *N_e_* in magnitude. It also breaks down when *t* is very small (as can be seen in the lower left panel for *t *=* *2, where the integration approximation actually becomes less accurate than the infinite population model). This is a consequence of the use of a continuous integration in Equation (6), which underestimates the total tree length when the number of discrete generations is small. However, in this small *t* regime, the recursion solution, which is most accurate, can also be easily evaluated due to the need for only few recursion steps.

### Extension to alternative crossing schemes

Our analytical approach for calculating E[D] is straightforward to extend to variations of the experimental design, such as alternative crossing schemes. The key parameters that need to be ascertained for a given design are the rate ρ(i) at which new ancestry breakpoints are generated per bp in the gametes that will make up the individuals in generation *i*, together with E[x(i)], the expected number of ancestral lineages present in the sample’s genealogy in that generation. The expected mapping resolution is then given by a direct generalization of Equation (3):

(10) $$E[D]=\frac{1}{\sum_{i=2}^{t}\rho (i)E[x(i)]}.$$

In the standard BSA design, we had ρ(2)=r and ρ(i>2)=r/2, with E[x(i)] calculated recursively by Equation (2) using the coalescence effective size *N_e_* of the interbreeding population. In the following, we will illustrate how this approach can be applied to two modifications of the standard BSA design.

**Fig. 6.**
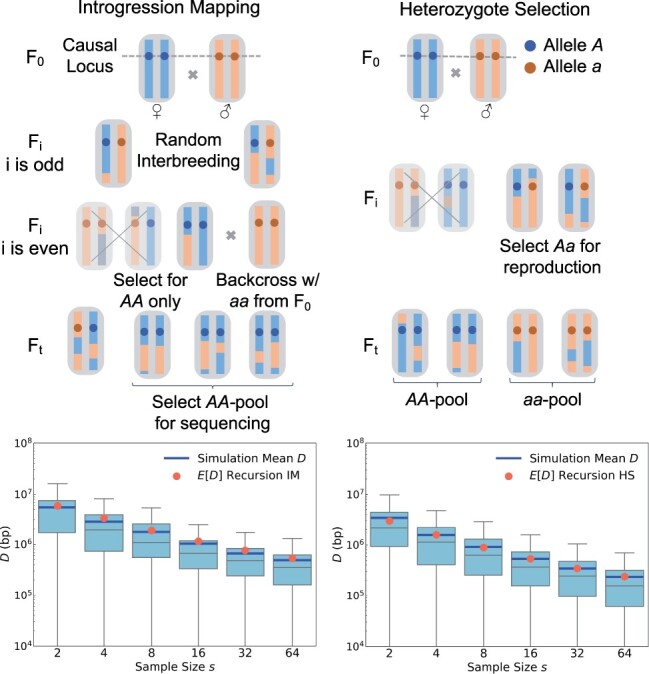
Extension of our theory to two different crossing schemes. In the IM scheme shown in the left panels, *AA* homozygotes are selected in even generations and then backcrossed to the *aa* founding strain. The resulting offspring are interbred without selection in odd generations. At the end of the experiment, *AA* homozygotes are sequenced. The bottom-left panel shows that the distributions of *D* values in simulated IM experiments conform well to our analytical predictions (see *Materials and methods*). The HS scheme shown in the right panels is similar to the standard BSA design, except that only *Aa* heterozygotes are allowed to reproduce in every generation. Our theory again accurately predicts the expected mapping resolution under this design (bottom-right panel).

The first approach is IM for a monogenic trait, illustrated in Fig. 6 (left). Here, *AA* homozygotes are selected in every even generation of the experiment (the approach thus relies on our ability to do so effectively). These individuals are then backcrossed to the *aa* parental strain. The resulting offspring are interbred without selection in every odd generation, after which the cycle starts anew. At the end of the experiment, *s* individuals of genotype *AA* are selected and sequenced. Their genomes should then resemble the *AA* strain across a genomic region that surrounds the causal QTL, while resembling the *aa* strain throughout the rest of the genome.

During the odd generations of an IM experiment, all individuals should be *Aa* heterozygotes at the QTL, and thus carry one red and one blue ancestry segment across some region surrounding it. These segments will become shorter and shorter due to recombination events as the experiment progresses. The rate at which new ancestry breakpoints are created close to the QTL, during the odd generations, should therefore be twice that of a standard BSA experiment, while it will be zero in all even generations (when all surviving individuals will be *AA* homozygotes at the QTL). When averaged over the whole experiment, new ancestry breakpoints in the vicinity of the QTL should hence arise at a rate of r/2, similar to the BSA design.

However, due to the selection step for *AA* homozygotes in the even generations, the coalescence rate in the IM design should be higher as compared to a standard BSA design with an interbreeding population of comparable size, given that only 1/4 of the population should be *AA* homozygotes. Thus, the value of *N_e_* will need to be adjusted in Equation (2). A reasonable approximation would be to use the harmonic mean between odd and even generations, yielding $N_{e}=0.4N\prime$, where N′ is the coalescence effective population size of the interbreeding population in a scenario where no selection and introgression would be performed. Figure 6 confirms that this approach produces an accurate analytical prediction for the expected mapping resolution in an IM experiment.

This example illustrates how our theory can help evaluate the expected performance of alterations to an experimental design. For the IM design in particular, the fact that ρ(i) should be comparable to a standard BSA design when averaged over the entire experiment, while *N_e_* should be smaller, suggests that an IM design for a monogenic trait should generally have lower resolution than BSA, confirming previous simulation results (Pool 2016). Yet, there may be other advantages of IM. For example, this design ensures that *A* and *a* alleles are kept at 50% frequency throughout the experiment, thereby eliminating any potential effects of drift or selection at the QTL that could exist in a standard BSA design.

The second alternative design we want to discuss is HS for a monogenic trait, illustrated in Fig. 6 (right). In this approach, only *Aa* heterozygotes are selected for reproduction in every generation (again assuming that we can do so effectively). This should double the rate of ancestry breakpoint generation in the vicinity of the QTL as compared to a standard BSA design, so that ρ(i)=r for all generations i≥2. However, the effective population size will again be reduced due to the selection step. Here, a reasonable approximation should be that *N_e_* is about 1/2 of that in a standard BSA design with an interbreeding population of comparable size, given that about half of the population are expected to be *Aa* heterozygotes at any point. Our simulations confirm that this approach again produces an accurate analytical prediction for the expected mapping resolution in an HS experiment (Fig. 6).

In principle, due to the higher rate of ancestry breakpoint generation, the HS design could therefore yield a mapping resolution for a monogenic trait that is up to two times better than a standard BSA design, as long as this is not outweighed by the concomitant reduction in *N_e_*. Note that, as with IM, the HS design maintains the frequency of *A* and *a* alleles at 50%.

### Pooled sequencing data

Our calculations have so far assumed full sequence information for all sampled chromosomes. The sequencing data in an actual BSA experiment, however, will often be comprised of short reads from pooled samples (Schlötterer et al. 2014). For such “Pool-seq” data, the specific set of chromosomes sequenced at any given position will thus vary along the genome, unless the sequencing coverage level is so high that each chromosome is covered by at least one read at most positions. This raises an important practical question: for a BSA experiment with Pool-seq data, is the number of sampled chromosomes (2*s*) or the sequencing coverage level (*C*) the more critical factor in determining mapping resolution?

Our calculations make a clear prediction. Since mapping resolution is ultimately limited by the ancestry breakpoints present in the sampled chromosomes, sample size will be the key limiting factor. In larger samples, there is simply a better chance to capture more breakpoints that are closer to the QTL. In fact, even when coverage is low compared to sample size, it may still be possible to achieve a mapping resolution close to what would be predicted by our equations for the given sample size. Consider, for example, two SNPs that are both close to the QTL yet separated from each other by a distance larger than the typical read length. In the Pool-seq data, we will then likely find different sets of chromosomes being captured by reads at each SNP. Each such SNP can therefore provide another chance to observe a read with ancestry from the opposite strain. As long as the read length is much shorter than the expected mapping resolution, this provides a large number of trials to detect alternative ancestries as we move away from the QTL, which could make up for the limited number of chromosomes sequenced at each individual locus.

To test this prediction, we simulated BSA experiments with either full sequencing data of a sample of size 2s=40, or Pool-seq data of *C *=* *40× coverage from a much larger sample of size 2s=400. We denote sample size here by 2*s* instead of *s*, since we want to compare a coverage equivalent to the number of chromosomes sequenced at each position in the full sequencing approach. Note, however, that generating full sequencing data for a sample of 40 chromosomes would actually require an overall sequencing coverage substantially higher than 40× with current sequencing technologies.

**Fig. 7.**
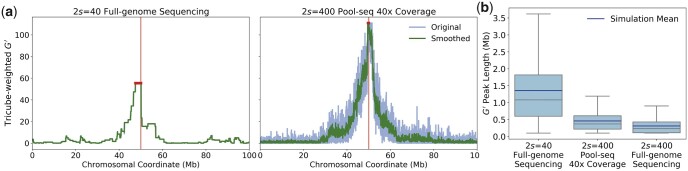
Full genome sequencing vs Pool-seq. a) G′ statistics estimated in two simulated BSA experiments that modeled a single QTL at the center of a 100-Mb-long chromosome, with *N_e_* = 100, *t *=* *10, and r=1e−8. The left plot shows full genome sequencing of a sample of size 2s=40. The right plot shows Pool-seq with 40× coverage of a sample of size 2s=400. The peak lengths of G′ were 2.56 Mb (left) and 0.35 Mb (right) in these two simulations, respectively. b) The box plots show the distributions of G′ peak lengths estimated across 1,000 simulation runs under each of three experimental setups: full-genome sequencing of samples of size 2s=40 (left), Pool-seq with 40× coverage of samples of size 2s=400 (center), and full-genome sequencing of samples of size 2s=400 (right). Remarkably, the Pool-seq experiment with C=40x and 2s=400 yields a resolutions that is almost as good as the full-sequencing experiment with 2s=400, despite having less than 10% of chromosomes actually genotyped at each locus, on average. Note that the lower whiskers of the three box plots are all at approximately 100 kb, specifying the maximally achievable resolution in our simulations given the smoothing procedure together with the fact that G′ was estimated from marker SNP placed at equidistant intervals of 10 kb along the chromosome.

Figure 7a shows that the Pool-seq approach can indeed achieve a much higher resolution than the full sequencing approach, despite a comparable number of chromosomes sequenced per site. One can also see how this is a result of the stochastic nature in which Pool-seq captures reads from different chromosomes as one moves along the sequence, which generates noise in the G′ curve. The QTL can be more precisely localized in the Pool-seq approach because due to the much larger sample size, the region around the QTL in which one never observes reads from both parental strains in the same pool is much shorter as compared to the G′ peak in the full sequencing approach with the smaller sample size.

This result is not just observed in one specific simulation run, but holds more generally for the distribution of peak lengths for the G′ statistic, estimated over 1,000 simulations (Fig. 7b). We find that the average peak length for the Pool-seq approach with 2s=400 and 40× is almost 10 times shorter than for the full sequencing approach with 2s=40. In fact, the Pool-seq approach yields a resolution that is just slightly worse than that of a full sequencing approach of the whole sample of size 2s=400, which would require substantially more sequencing effort. Interestingly, our results suggest that longer reads in a Pool-seq approach would actually perform worse in such situations, as this would increase the distance over which the set of sequenced chromosomes would remain correlated.

### Impact of phenotyping inaccuracy

The arguably most unrealistic assumptions made in our calculations is the lack of contamination of the segregant pools by alternative alleles, which would require a perfect ability to assess QTL genotype by phenotype. In practice, factors such as incomplete heritability or heterozygotes showing a similar phenotype as one of the homozygotes will likely result in some level of contamination in most experiments. We would expect that such contamination can decrease the actual mapping resolution as compared to an experiment with perfect phenotyping.

**Fig. 8.**
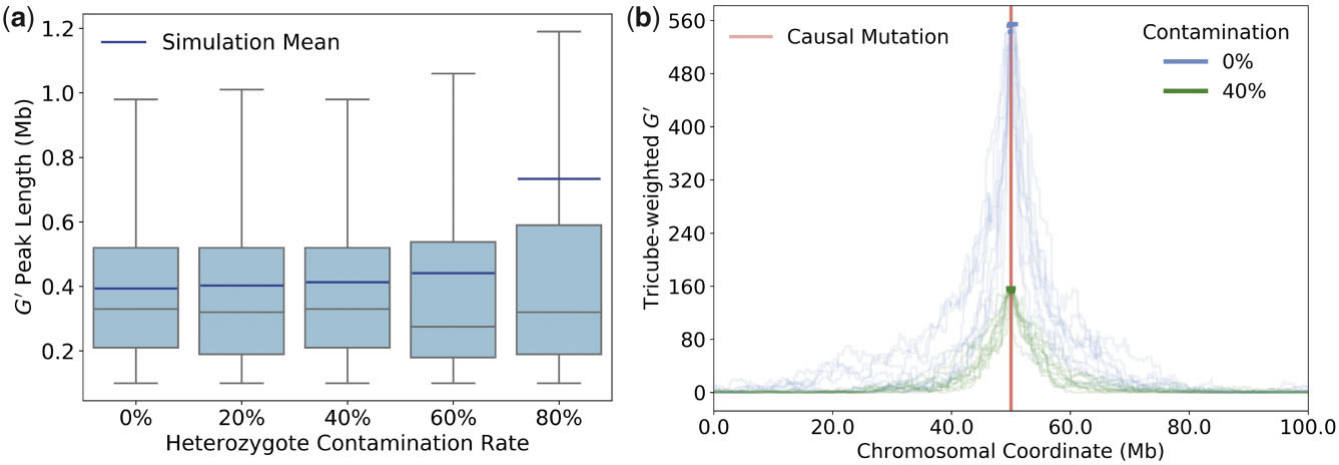
The impact of heterozygote contamination on BSA experiments. a) Box plots show the distributions of G′ peak lengths as a function of the heterozygote contamination rate in the segregant pools, estimated from 1,000 simulation runs for each rate. We assumed equal percentages of heterozygotes in both pools, with no contamination by homozygotes of the alternative allele. Mean G′ peak lengths are surprisingly robust to contamination levels as high as 60%. Note that simulation means are systematically higher than medians, especially for the highest tested contamination level of 80%, indicating that mean values are likely driven by long tails of the distributions. As in Fig. 7b, the lower whiskers always extend to the maximally achievable resolution of approximately 100 kb given our smoothing procedure and distance between marker SNPs. b) The curves show tricube weighted G′ values for 10 simulation runs each for a scenario without heterozygote contamination and a scenario with a heterozygote contamination rate of 40%. Bold segments of the curves specify the identified peaks. Contamination lowers the average height of the G′ peaks but their average length is much less affected. For the 0% scenario, peak lengths ranged from 0.10 to 0.83 Mb in our simulations, as compared to 0.12–1.02 Mb for the 40% scenario.

To examine the magnitude of this effect, we compared the distributions of G′ peak lengths in simulated BSA experiments with different levels of contamination by alternative alleles (Fig. 8a). Intriguingly, even when 40% of individuals in each pool were heterozygotes, this still increased the mean peak length only marginally (by just a few percent). Thus, the results we derived for the expected mapping resolution under the assumption of no contamination should still be reasonably accurate in these scenarios.

The reason for this apparent robustness to contamination by alternative alleles can be seen in Fig. 8b, where G′ curves are shown for individual simulation runs with 0% vs 40% contamination by heterozygotes in each pool. While higher contamination rate clearly decreases the height of the G′ peaks around the QTL, peak widths are much less affected. This makes sense given the definition of G′, which essentially measures allele-frequency differences between the segregant pools. Even with 40% contamination in each pool, allele frequencies at the causal locus should still segregate at a ratio of 20:80 between the two pools, as compared to a ratio of 0:100 with no contamination, and a ratio of 50:50 for the genomic background. As one moves away from the QTL, this signal will decay at a similar rate as for the scenario without contamination. Thus, as long as contamination is not yet so high that it becomes difficult to discern the peak from background noise, the expected peak size will remain similar. Note that the same line of reasoning, in principle, should also hold for the presence of heterozygous SNPs in the two founding strains.

### Comparison with experiments

To further validate the accuracy of our mathematical predictions, we compared the mapping resolution predicted by Equation (10) with the empirical resolution achieved in two recent BSA experiments. The first experiment was a mapping study of the genetic markers for intramuscular fat in Wagyu cattle (Zhu et al. 2021), which identified a genomic candidate region of length ∼5 Mb (between positions 24.8 and 29.6 Mb on chromosome 23). In this modified IM experiment, female Chinese Waygu beef cattle were hybridized with sperm from a single male Japanese Wagyu bull, and the progeny population was repeatedly back-crossed with sperm from the same bull until the *F*_3_. A sample of *s *=* *13 cattle were collected from the *F*_3_ and their genomes were sequenced at an average of 10× coverage per individual.

The average recombination rate across 29 bovine chromosomes in beef cattle has been estimated at 1.23* *cM/Mb (i.e. r=1.23e−8) (Weng et al. 2014). The effective population size of the experiment is unknown but can be roughly calculated as $N_{e}=4N_{f}N_{m}/(N_{f}+N_{m})$, where *N_m_* = 1 is the number of male individuals throughout the whole experiment and *N_f_* is the number of female individuals randomly selected in each generation. Since $N_{f}\gg N_{m}$, a reasonable approximation would be $N_{e}\approx 4$. Besides, under the given parameters, even when *N_e_* is varied over a broad range it will only have a minor impact on the predicted mapping resolution. In particular, for the given experimental design with *t *=* *3, *s *=* *13, and r=1.23e−8, Equation (10) predicts that the expected peak size should be E[D]=3.9 Mb for *N_e_* = 4, 2.2 Mb for *N_e_* = 100, and 2.1 Mb for *N_e_* = 1,500. Thus, the average overall peak length 2E[D] should be roughly 4−8 Mb even across this wide range of *N_e_* values. Given the generally high variance in peak lengths, this is in good agreement with the observation of a 5-Mb-long genomic region detected in the experiment.

The second experiment was a mapping study for pyrethroid resistance in the house fly *Musca domestica* (Freeman 2020). In this experiment, flies from a susceptible male strain and a resistant female strain were crossed and the progeny interbred until the *F*_6_, with the interbreeding population ranging in size from ∼5,000 flies in the *F*_2_ to ∼9,000 flies from the *F*_3_ on. The effective population size is again unknown, but should presumably be somewhere within the range of 1,000−5,000. At the end of the experiment, two pools of 100 completely resistant and 100 completely susceptible flies were selected, and each pool was sequenced with 30−40x coverage using a Pool-seq approach. Assuming a standard BSA design with an average recombination rate for house flies of r=0.74e−8 (Feldmeyer et al. 2010), a sample size of *s *=* *200, and *t *=* *6 generations of interbreeding, our calculations predict an expected genomic resolution between E[D]=0.14 Mb for *N_e_* = 1,000, and E[D]=0.12 Mb for $N_{e}=5,000$.

The highest peak in this study was located on chromosome 3, with the maximum G′ value observed at position 4,459,036 bp. This falls inside the *Vssc* gene, which harbors both the *kdr* and *skdr* mutations that have previously been identified to confer resistance to pyrethroids. In particular, the position of the maximum G′ value was 87 kb from the *kdr* mutation and 82 kb from the *skdr* mutation. Both mutations were present in 100% of the reads for the resistant pool, yet observed at only low frequency in the susceptible pool. Again, these numbers are in good agreement with our mathematical prediction of an expected mapping resolution of roughly 100−150 kb for the given experimental parameters.

## Discussion

BSA has become an increasingly popular technique for mapping the genetic basis of phenotypic traits. Previous studies have used simulations to study how the genomic resolution of BSA is affected by key experimental parameters such as sample size and number of generations of interbreeding (Pool 2016). However, a truly quantitative understanding has so far remained elusive. In this study, we were able to derive a mathematical prediction for the expected mapping resolution of a BSA experiment. We have further demonstrated how our framework can be extended to modifications of the experimental design, such as IM or selection for heterozygotes.

Our approach is based on the insight that the mapping resolution of a BSA experiment is ultimately limited by the length of the genomic region surrounding the QTL in which all sequences in each sampled pool still share the ancestry of the respective parental strain. This region is delimited by the two closest ancestry breakpoints observed upstream and downstream of the QTL. We modeled the occurrence of such breakpoints by a Poisson process along the chromosome, with its rate determined by two factors: the expected length of the sample’s genealogy at any given genomic position, and the expected rate at which new ancestry breakpoints were generated along this genealogy from the ancestors to the sample. Both factors combine to determine the expected mapping resolution according to Equation (10).

Our solution sheds light on the possible avenues for improving mapping resolution. First, the rate of ancestry breakpoint generation could be increased. While this rate is obviously bounded by the recombination rate of the organism, only recombination events in individuals that carry ancestry segments from both parental strains at the crossover location actually generate new ancestry breakpoints. Thus, one could seek to increase the frequency of such individuals; this is the rationale behind the “heterozyogte selection” strategy we discussed above. Second, the length of the sample’s genealogy could be increased. In principle, this could be achieved by including more generations of inbreeding, using a larger sample size (and thus a larger number of phenotyped individuals), or achieving a lower coalescence rate during the experiment (which would typically require a larger population size during interbreeding). Exactly how these parameters play out will depend on the specific experimental setup.

In Equation (7), we provided an approximate solution for the maximal mapping resolution of a standard BSA experiment. This solution requires specification of the coalescence effective population size (*N_e_*) of the interbreeding population that determines the pairwise coalescence rate in the genealogy of the sample. In practice, the value of *N_e_* will typically be smaller than the actual number of individuals present in the interbreeding population, especially when there is high variance in offspring number among individuals (Charlesworth 2009). While various methods have been developed for inferring *N_e_* of experimental populations (Williamson and Slatkin 1999; Anderson 2005; Jónás et al. 2016; Liu et al. 2019), such inference may be nontrivial, and it may thus be unclear how to choose the appropriate value for this parameter; at a minimum, however, the population size of the interbreeding population constitutes an upper bound for *N_e_*. Our analysis suggests that *N_e_* should generally be kept as large as possible throughout the experiment to optimize mapping resolution.

For the sake of mathematical tractability, we have focused on a rather simplistic model of a trait controlled by a single QTL. For traits determined by multiple loci, overlap between signals could become a problem (Pool 2016), and it may no longer be possible to select for individuals that are homozygous at all QTLs. In such cases, we expect that our results can only provide a lower bound to the achievable mapping resolution.

Our approach further assumed that recombination rate is uniform along the chromosome, but it would be rather straightforward to incorporate nonuniform recombination rates. In particular, recombination events would then need to be modeled by an inhomogeneous Poisson process in Eq. (3), so that *R*, and thus also E[D], would become a function of genomic position. Intuitively, one would expect higher mapping resolution in regions of higher recombination rate, and vice versa.

Another simplification of our modeling is perfect sequencing data, while any real-world experiment will likely suffer from some level of imperfect estimation of pool allele frequencies due to sequencing errors or low coverage. This should increase “noise” in summary statistics such as G′, possibly making it more difficult to delimit or even identify individual peaks when they no longer stand out against the fluctuations observed along the genomic background. Such noise could be particularly problematic when the segregant pools also have high levels of contamination by alternative alleles, as we have shown that this can lower the expected height of the peaks (Fig. 8).

In the early days of molecular genetics, the precision one could hope to achieve in a mapping experiment was typically limited by the ability to genotype a sufficient number of individuals at a sufficiently dense set of marker loci. With the sequencing revolution, this constraint has fundamentally shifted. Today, it is often feasible to obtain whole-genome sequencing data for samples of several hundreds or even thousands of individuals. Consequently, it is becoming more relevant to understand which other factors fundamentally limit mapping resolution under a given experimental design. By providing a mathematical prediction for the expected mapping resolution of a BSA experiment, based on coalescence theory, we were able to shed light on how individual parameters combine, qualitatively and quantitatively, to place a fundamental limit on mapping resolution. From an experimentalists’ perspective, another advantage of our mathematical approach over existing simulation-based alternatives is not needing to run large computationally intensive simulations for predicting the expected mapping resolution. We hope that these results can not only help scientists to set realistic expectations for the power of their planned experiments, but also to identify which strategies would allow them to optimize their study design most efficiently and economically. Finally, we hope that the conceptual approach that underlies our calculations can be extended to other genetic mapping strategies.

## Data availability

The code used to generate the simulated data and analyze the simulation results can be found at https://github.com/runxi-shen/Predict-Genomic-Resolution-of-BSA.
